# Monoclonal Antibody for Reducing Memory and Learning Problems in Schizophrenia

**Published:** 2018

**Authors:** Shahin Akhondzadeh

**Affiliations:** Psychiatric Research Center, Roozbeh Hospital, Tehran University of Medical, Sciences, Tehran, Iran

Schizophrenia is a chronic debilitating psychiatric illness that accounts for a significant portion of the burden caused by mental illnesses worldwide. Primary negative symptoms of schizophrenia are not secondary to extrapyramidal, depressive or positive symptoms 1,2. Negative symptoms are the core features of the illness which are associated with long-term functional disability and poor outcome 1,3. These symptoms include deficits in social and emotional functioning, blunted affect and lack of spontaneity. There is a growing body of evidence for the role of inflammation and immune system dys-regulation in psychiatric disorders 4. Although the precise pathophysiology of schizophrenia is not completely known, a number of recent studies support the probable pathologic role of immunologic dysfunction in this disorder. Assessing serum cytokine levels such as interleukin 1 (IL-1), IL-2, IL-6, and chemokine CCL11 in schizophrenic patients demonstrates profound alterations compared to healthy matched controls 4. Furthermore, increased cyclooxygenase-2 (COX-2) expression as well as prostaglandin E2 production in schizophrenia, are among other postulated etiologies supported by recent studies 4. On the other hand, it has been shown that immune response imbalance is associated with decreased activity of indoleamine 2, 3-dioxygenase enzyme which subsequently leads to accumulation of kynurenic acid, an endogenous antagonist of glutamate N-methyl-D-aspartate (NMDA) receptor. Compared with anitinflammatory agents like celecoxib and NAC, monoclonal antibodies also have more potent anti-inflammatory properties. Indeed, COX-2 inhibitors and N-acetylcysteine have moderate efficacy in treatment of schizophrenia and autism 1,2,5. British scientists have begun testing a radically new approach to treating schizophrenia based on emerging evidence that it could be a disease of the immune system. Evidence for prenatal and premorbid immune risk factors for the development of schizophrenia in the offspring is highlighted 6,7. Then key evidence for immune dysfunction in patients with schizophrenia is considered. A collaboration between the Medical Research Council (MRC) and King’s College London, is based on emerging evidence that schizophrenia may be an immune disease. The drug, natalizumab, works by targeting microglia, a type of immune cell residing in the brain which are thought to be overactive in people at risk of developing schizophrenia 6,7.
